# Supplementary material for: Content-rich biological network constructed by mining PubMed abstracts
Source: BMC Bioinformatics. 2004 Oct 8;5:147. doi: 10.1186/1471-2105-5-147 (PMC528731; doi:10.1186/1471-2105-5-147)
Supplement: Additional File 2 — The original results of the above study (non-essential files are deleted to keep the file size under the limit set by BMC bioinformatics). [file 1471-2105-5-147-S2.bz2 › chilibotAdditionalFile2/dip20/611ID10580496E10994/html/ATPE_ATPB.html]

 


 **ATPE** and **ATPB** 
  
Found 26 abstracts in PubMed, retrieved 20.  
 

 What does Google say? 
 PDF only 
| .edu only 

---

**Interactive relationship** (e.g. stimulation, inhibition, etc)

**Neutral relationship**- Although the coding site of  **atpE**  cannot be demonstrated by heterologous Southern hybridizations, a 417 bp reading frame 3 to  **atpB**  was identified as the gene  **atpE**  by sequence similarities with  **atpE**  genes from other sources.  Ref: 1535802 Plant Mol Biol, 1992
- The 5 end of the  **atpE**  specific transcript has been mapped 430 431 nt upstream of the  **atpE**  translation initiation site, within the coding region of the  **atpB**  gene.  Ref: 7859310 Curr Genet, 1994

**Non-interactive relationship** (e.g. studied together, co-existance, homology, etc.)

- We have cloned and sequenced the genes  **atpB**  and  **atpE** , coding for CF1 subunits beta and epsilon, respectively, of the chloroplast genome of the brown alga Dictyota dichotoma.  Ref: 1535802 Plant Mol Biol, 1992
- Two independent intercistronic RNA processing events and 11 splicing reactions lead to the accumulation of the mature petB,  **atpB**  and  **atpE**  monocistronic mRNAs.  Ref: 7923415 Curr Genet, 1994
- The affinities of binding of 30 S ribosomal subunits showed the relationship  **atpE**  greater than  **atpB**  greater than atpG.  Ref: 2693739 J Mol Biol, 1989
- The genes encoding cytochrome b6 of the chloroplast cytochrome b6 f complex petB and the ATP synthase CF1 beta subunit  **atpB**  and epsilon subunit  **atpE**  were identified on the EcoD fragment of the Euglena gracilis chloroplast genome.  Ref: 7923415 Curr Genet, 1994
- The sequence starts from one end JLA of the large single copy region and encompasses genes for 21 tRNAs, six ATPase subunits atpA,  **atpB** ,  **atpE** , atpF, atpH and atpI, two photosystem I polypeptides psaA and psaB, four photosystem II polypeptides psbA, psbC, psbD and psbG, five ribosomal proteins rps2, rps4, rps7, rps 12 and rps14, and three RNA polymerase subunits rpoB, rpoC1 and rpoC2.  Ref: 2974085 J Mol Biol, 1988
- The  **atpB**  and  **atpE**  genes encode beta and epsilon subunits, respectively, of chloroplast ATP synthase and are co transcribed in the plant species so far studied.  Ref: 7859310 Curr Genet, 1994
- No genes for photosynthetic functions have been found, except for sequences encoding six subunits of the ATP synthase atpA,  **atpB** ,  **atpE** , atpF, atpH, and atpI.  Ref: 12111556 Mol Genet Genomics, 2002
- Promoter leader regions of the  **atpB** ,  **atpE** , rps4, and rps7 genes representing the diversity of chloroplast SD like sequences were fused to aadA and uidA reporter genes encoding spectinomycin resistance and GUS activity respectively.  Ref: 9520261 Mol Gen Genet, 1998
- This region contains in the following order genes for the cytochrome b6 f complex subunit V petG, tRNA Val trnV, tRNA Met trnM, the epsilon and beta subunit of the chloroplast ATP synthase  **atpE**  and  **atpB**  and the large subunit of ribulose bisphosphate carboxylase oxygenase Rubisco.  Ref: 1552899 Mol Gen Genet, 1992
- This gene organization differs from that of land plants in which  **atpB**   **atpE**  form a discistronic operon, and petB is within the psbB ycf8 psbH petB petD operon.  Ref: 7923415 Curr Genet, 1994
- The ninth KpnI fragment about 4.0 kb of broad bean chloroplast DNA contains the entire chloroplast  **atpE**  gene and  **atpB**  gene.  Ref: 12168017 Sheng Wu Hua Xue Yu Sheng Wu Wu Li Xue Bao Shanghai < MedlineTA>Sheng Wu Hua Xue Yu Sheng Wu Wu Li Xue Bao Shanghai , 1994
- **atpB**   **atpE** .  Ref: 1400453 J Biol Chem, 1992
- A nucleotide sequence of 7556 bp contained structures similar to those of ndhK, ndhC, trnV, trnM,  **atpE** ,  **atpB** , rbcL, trnR and accD.  Ref: 8604330 Nucleic Acids Res, 1996
- An  **atpE**  specific promoter within the coding region of the  **atpB**  gene in tobacco chloroplast DNA.  Ref: 7859310 Curr Genet, 1994
- Three chloroplast genes,  **atpB** ,  **atpE**  and trnM, that respectively encode the beta and epsilon subunits of ATP synthase and tRNA met, were sequenced.  Ref: 1388032 Jpn J Genet, 1992
- The petB  **atpB**   **atpE**  genes are cotranscribed as a tricistronic operon.  Ref: 7923415 Curr Genet, 1994
- The control of the atp genes expression rates has been shown to be exercised primarily at the level of translational initiation, but how is this achieved in molecular terms ? In order to study the interactions of 30 S ribosomal subunits with specifically the translational initiation regions TIRs of  **atpB** ,  **atpE**  and atpG, restriction fragments bearing these TIRs were excised from the atp operon and cloned into an SP6 promoter transcription vector.  Ref: 2693739 J Mol Biol, 1989
- The genes for the five subunits were present in the order atpI subunit I  [ **ATPB** ] ,  **atpB**  subunit a,  **atpE**  subunit c, atpX subunit b, and atpF subunit b.  Ref: 9818357 Arch Microbiol, 1998
- We estimate that the functional half life of  **atpB**  is slightly over one half that of  **atpE**  and the other atp genes, while atpI is at least two times less stable than  **atpB** .  Ref: 8679701 Biochim Biophys Acta, 1996
- As in cyanobacteria, the  **atpE**  gene does not overlap the preceding gene  **atpB** .  Ref: 1535802 Plant Mol Biol, 1992
- Eight genes atpI,  **atpB** ,  **atpE**  1,  **atpE**  2,  **atpE**  3, atpF, atpH, and atpA upstream of and contiguous with the previously described genes atpG, atpD, and atpC were cloned from chromosomal DNA of Acetobacterium woodii.  Ref: 10567365 J Biol Chem, 1999
- The C. thermoaceticum atp operon contained nine genes arranged in the order atpI i,  **atpB**  a,  **atpE**  c, atpF b, atpH delta, atpA alpha, atpG gamma, atpD beta, and atpC epsilon.  Ref: 9171425 J Bacteriol, 1997
- A range of translational initiation regions TIR was created by combining synthetic DNA fragments derived from the  **atpB**   **atpE**  intercistronic sequence of Escherichia coli with the cDNA sequence encoding mature human interleukin 2 IL 2, the E. coli fnr gene, or an fnr lacZ gene fusion.  Ref: 2475391 Gene, 1989
- The genes  **atpB**  and  **atpE** , encoding ATPase subunits beta and epsilon, respectively, are linked in atp2, separated by a 2 bp spacer.  Ref: 8219057 Plant Mol Biol, 1993
- The base substitution rate of the  **atpB**   **atpE**  trnM gene cluster was similar to that of the rbcL gene.  Ref: 1388032 Jpn J Genet, 1992
- The genes were organized in an operon with the gene order atpEBFHAGDC ; i.e., the order of  **atpE**  and  **atpB**  is reversed with respect to the more typical bacterial organization.  Ref: 10940012 J Bacteriol, 2000
- In Euglena, the petB  **atpB**   **atpE**  genes contain introns, including two twintrons, at eight different positions.  Ref: 7923415 Curr Genet, 1994
